# 4-Bromo-4′-(dimethyl­amino)stilbene

**DOI:** 10.1107/S1600536809020492

**Published:** 2009-06-06

**Authors:** Rodolfo Moreno-Fuquen, Alan R. Kennedy, Cristina Cordoba

**Affiliations:** a Departamento de Química – Facultad de Ciencias, Universidad del Valle, Apartado 25360, Santiago de Cali, Colombia; b WestCHEM, Department of Pure and Applied Chemistry, University of Strathclyde, 295 Cathedral Street, Glasgow G1 1XL, Scotland

## Abstract

In the title compound, C_16_H_16_BrN, the benzene rings are inclined to each other with a dihedral angle between their mean planes of 50.5 (3)° and the C=C bond adopts a *cis* conformation.

## Related literature

For background information on photonic materials, see: He *et al.* (2008 ▶). For related systems of stilbene, see: Moreno-Fuquen *et al.* (2008 ▶, 2009 ▶). For literature related to the synthesis, see: Maryanoff & Reitz (1989 ▶).
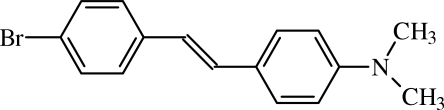


## Experimental

### 

#### Crystal data

C_16_H_16_BrN
*M* *_r_* = 302.21
Monoclinic, 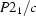

*a* = 14.804 (2) Å
*b* = 6.0962 (5) Å
*c* = 15.2106 (10) Å
β = 95.331 (9)°
*V* = 1366.8 (2) Å^3^
*Z* = 4
Mo *K*α radiation
μ = 2.99 mm^−1^
*T* = 123 K
0.38 × 0.25 × 0.10 mm

#### Data collection

Bruker APEXII CCD diffractometer
Absorption correction: multi-scan (*SADABS*; Sheldrick, 2002 ▶) *T*_min_ = 0.457, *T*_max_ = 0.749
8606 measured reflections
2986 independent reflections
1533 reflections with *I* > 2σ(*I*)
*R*_int_ = 0.064

#### Refinement

*R*[*F* ^2^ > 2σ(*F* ^2^)] = 0.075
*wR*(*F* ^2^) = 0.213
*S* = 0.99
2986 reflections
165 parameters
H-atom parameters constrained
Δρ_max_ = 0.94 e Å^−3^
Δρ_min_ = −0.54 e Å^−3^

### 

Data collection: *APEX2* (Bruker, 2007 ▶); cell refinement: *SAINT* (Bruker, 2007 ▶); data reduction: *SAINT*; program(s) used to solve structure: *SHELXS97* (Sheldrick, 2008 ▶); program(s) used to refine structure: *SHELXL97* (Sheldrick, 2008 ▶); molecular graphics: *ORTEP-3 for Windows* (Farrugia, 1997 ▶); software used to prepare material for publication: *PARST95* (Nardelli, 1995 ▶).

## Supplementary Material

Crystal structure: contains datablocks I, global. DOI: 10.1107/S1600536809020492/hg2518sup1.cif

Structure factors: contains datablocks I. DOI: 10.1107/S1600536809020492/hg2518Isup2.hkl

Additional supplementary materials:  crystallographic information; 3D view; checkCIF report

## Figures and Tables

**Fig. 1.**
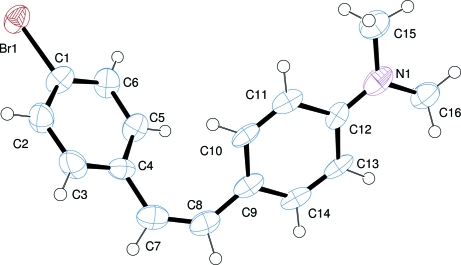
An ORTEP-3 (Farrugia, 1997) plot of the DMBS compound, with the atomic labelling scheme. The shapes of the ellipsoids correspond to 50% probability contours of atomic displacement and, for the sake of clarity, H atoms are shown as spheres of arbitrary radius.

## supplementary crystallographic information

### Comment

The present work is part of a structural study of molecular complexes based on the matrix of stilbene which can be used as non-linear optical material (He *et al.*, 2008). Our research group has developed the study of other related systems of stilbene (Moreno-Fuquen *et al.*, 2008; Moreno-Fuquen *et al.*, 2009). Though the present molecular system is centrosymmetric, information about its crystal structure is very important to the study of the general behavior of stilbenes because crystallographic information of stilbene systems is still rather small. The main objective of the present work is to present the molecular and crystal structure of the 4-dimethylamino-4'-bromostilbene (DMBS) and to analyse the conformational structure of the system. A perspective view of the molecule of the title compound, showing the atomic numbering scheme, is given in Fig. 1. The benzene rings ot the title structure are inclined to each other showing a dihedral angle between their mean planes of 50.5 (3)°. The phenyl rings are twisted out of the ethylene bond plane, and are defined by the the torsion angles C5—C4—C7=C8 and C7=C8—C9—C10. The dimethylamino group forms a dihedral angle of 8.6 (7)° with respect to its phenyl ring. The title molecule shows a torsion angle C4 C7 C8 C9 of 7.1 (15)° indicating the existence of a great repulsion between the aromatic rings. These values allow to define its conformation structure as *cis*. The title system does not observe the formation of intermolecular hydrogen bonds.

### Experimental

By means of Wittig reaction (Maryanoff & Reitz, 1989), the 4-dimethylamino-benzyl-triphenylphosphonium iodide was prepared. The title stilbene was obtained by the reaction of equimolar quantities of phosphonium salt and 4-bromo benzaldehyde (0.03 mol) in THF solution. The mixture was maintained with stirring under argon atmosphere. The reaction mixture was kept at 273 K and it was dropped with a solution of *tert*-butanol and potassium *tert*-butoxide. Crystals of medium quality but suitable for single-crystal X-ray diffraction were grown in chloroform. An attempt was made to improve the quality of the crystals without success. Thin layer chromatography (TLC) was used to confirm the structure of the individual compounds. IR spectra were recorded on a Shimadzu FT—IR 8400 spectrophotometer.

*N*-(*p*-chlorophenyl)maleimide. Yellow crystals; yield 60%; mp 354 (1) K. IR (KBr) 2884 cm^-1^ (C—H), 3433 cm^-1^ (=C—H), 1609 cm^-1^ (C=C), 815 cm^-1^ (C=Br).

### Refinement

All H-atoms were located from difference maps and then they were treated as riding atoms [C_aro_—H= 0.93 A° and C_sp3_—H= 0.96 A°, *U*_iso_(H)= 1.2*U*_eq_(C_aro_), *U*_iso_(H)= 1.5*U*_eq_(C_sp3_)).

### Crystal data

**Table:**

C_16_H_16_BrN	*F*(000) = 616
*M**_r_* = 302.21	*D*_x_ = 1.469 Mg m^−^^3^
Monoclinic, *P*2_1_/*c*	Melting point: 354(1) K
Hall symbol: -P 2ybc	Mo *K*α radiation, λ = 0.71073 Å
*a* = 14.804 (2) Å	Cell parameters from 2824 reflections
*b* = 6.0962 (5) Å	θ = 2.7–29.0°
*c* = 15.2106 (10) Å	µ = 2.99 mm^−^^1^
β = 95.331 (9)°	*T* = 123 K
*V* = 1366.8 (2) Å^3^	Slab, yellow
*Z* = 4	0.38 × 0.25 × 0.10 mm

### Data collection

**Table:**

Bruker APEXII CCD diffractometer	2986 independent reflections
Radiation source: fine-focus sealed tube	1533 reflections with *I* > 2σ(*I*)
graphite	*R*_int_ = 0.064
φ and ω scans	θ_max_ = 27.0°, θ_min_ = 2.7°
Absorption correction: multi-scan (*SADABS*; Sheldrick, 2002)	*h* = −18→18
*T*_min_ = 0.457, *T*_max_ = 0.749	*k* = −7→7
8606 measured reflections	*l* = −19→19

### Refinement

**Table:**

Refinement on *F*^2^	Primary atom site location: structure-invariant direct methods
Least-squares matrix: full	Secondary atom site location: difference Fourier map
*R*[*F*^2^ > 2σ(*F*^2^)] = 0.075	Hydrogen site location: inferred from neighbouring sites
*wR*(*F*^2^) = 0.213	H-atom parameters constrained
*S* = 0.99	*w* = 1/[σ^2^(*F*_o_^2^) + (0.1119*P*)^2^] where *P* = (*F*_o_^2^ + 2*F*_c_^2^)/3
2986 reflections	(Δ/σ)_max_ < 0.001
165 parameters	Δρ_max_ = 0.94 e Å^−^^3^
0 restraints	Δρ_min_ = −0.54 e Å^−^^3^

### Special details

**Table:**

Geometry. All e.s.d.'s (except the e.s.d. in the dihedral angle between two l.s. planes) are estimated using the full covariance matrix. The cell e.s.d.'s are taken into account individually in the estimation of e.s.d.'s in distances, angles and torsion angles; correlations between e.s.d.'s in cell parameters are only used when they are defined by crystal symmetry. An approximate (isotropic) treatment of cell e.s.d.'s is used for estimating e.s.d.'s involving l.s. planes.
Refinement. Refinement of *F*^2^ against ALL reflections. The weighted *R*-factor *wR* and goodness of fit *S* are based on *F*^2^, conventional *R*-factors *R* are based on *F*, with *F* set to zero for negative *F*^2^. The threshold expression of *F*^2^ > σ(*F*^2^) is used only for calculating *R*-factors(gt) *etc*. and is not relevant to the choice of reflections for refinement. *R*-factors based on *F*^2^ are statistically about twice as large as those based on *F*, and *R*- factors based on ALL data will be even larger.

### Fractional atomic coordinates and isotropic or equivalent isotropic displacement parameters (Å^2^)

**Table:**

	*x*	*y*	*z*	*U*_iso_*/*U*_eq_	
Br1	0.36239 (6)	−0.41740 (14)	0.35579 (5)	0.0487 (4)	
N1	−0.0563 (6)	0.4693 (11)	0.6162 (5)	0.055 (2)	
C1	0.3671 (6)	−0.2184 (13)	0.4545 (5)	0.043 (2)	
C2	0.4151 (6)	−0.2818 (16)	0.5312 (5)	0.055 (2)	
H2	0.4433	−0.4217	0.5367	0.066*	
C3	0.4210 (6)	−0.1319 (16)	0.6019 (5)	0.055 (2)	
H3	0.4562	−0.1695	0.6552	0.066*	
C4	0.3768 (5)	0.0699 (13)	0.5960 (5)	0.0394 (19)	
C5	0.3297 (6)	0.1216 (13)	0.5151 (5)	0.046 (2)	
H5	0.3014	0.2613	0.5089	0.055*	
C6	0.3215 (6)	−0.0195 (14)	0.4429 (5)	0.050 (2)	
H6	0.2871	0.0176	0.3892	0.061*	
C7	0.3859 (6)	0.2246 (15)	0.6700 (5)	0.050 (2)	
H7	0.4442	0.2300	0.7017	0.060*	
C8	0.3242 (6)	0.3591 (14)	0.6993 (5)	0.047 (2)	
H8	0.3483	0.4566	0.7442	0.056*	
C9	0.2261 (6)	0.3859 (12)	0.6758 (4)	0.042 (2)	
C10	0.1699 (6)	0.2157 (13)	0.6391 (4)	0.039 (2)	
H10	0.1963	0.0776	0.6278	0.047*	
C11	0.0804 (6)	0.2435 (12)	0.6198 (5)	0.041 (2)	
H11	0.0458	0.1253	0.5936	0.049*	
C12	0.0344 (6)	0.4455 (11)	0.6371 (5)	0.0369 (18)	
C13	0.0914 (6)	0.6088 (12)	0.6775 (4)	0.042 (2)	
H13	0.0663	0.7459	0.6924	0.050*	
C14	0.1825 (6)	0.5720 (12)	0.6956 (5)	0.043 (2)	
H14	0.2178	0.6859	0.7242	0.052*	
C15	−0.1121 (6)	0.3046 (14)	0.5665 (6)	0.054 (2)	
H15A	−0.0868	0.2745	0.5105	0.081*	
H15B	−0.1742	0.3600	0.5547	0.081*	
H15C	−0.1127	0.1692	0.6012	0.081*	
C16	−0.1007 (6)	0.6780 (14)	0.6312 (6)	0.055 (2)	
H16A	−0.0761	0.7393	0.6880	0.082*	
H16B	−0.1661	0.6543	0.6319	0.082*	
H16C	−0.0898	0.7805	0.5837	0.082*	

### Atomic displacement parameters (Å^2^)

**Table:**

	*U*^11^	*U*^22^	*U*^33^	*U*^12^	*U*^13^	*U*^23^
Br1	0.0677 (7)	0.0433 (5)	0.0349 (4)	0.0116 (4)	0.0043 (3)	−0.0004 (4)
N1	0.080 (7)	0.033 (4)	0.053 (4)	0.003 (4)	0.010 (4)	−0.008 (3)
C1	0.068 (6)	0.037 (5)	0.025 (4)	0.000 (4)	0.010 (4)	0.008 (3)
C2	0.057 (6)	0.069 (6)	0.038 (5)	0.022 (5)	0.001 (4)	0.001 (5)
C3	0.047 (6)	0.085 (7)	0.032 (4)	0.014 (5)	−0.001 (4)	0.005 (5)
C4	0.037 (5)	0.051 (5)	0.030 (4)	−0.010 (4)	0.002 (3)	−0.001 (4)
C5	0.061 (6)	0.043 (5)	0.036 (4)	0.003 (4)	0.014 (4)	0.006 (4)
C6	0.064 (6)	0.062 (6)	0.025 (4)	0.019 (5)	0.004 (4)	0.015 (4)
C7	0.058 (6)	0.059 (6)	0.031 (4)	−0.012 (5)	0.000 (4)	0.008 (4)
C8	0.066 (7)	0.052 (5)	0.024 (4)	−0.011 (5)	0.011 (4)	0.001 (4)
C9	0.075 (7)	0.030 (5)	0.020 (3)	−0.006 (4)	0.007 (3)	0.004 (3)
C10	0.065 (6)	0.036 (5)	0.019 (3)	−0.008 (4)	0.014 (3)	−0.007 (3)
C11	0.068 (7)	0.031 (4)	0.026 (4)	−0.017 (4)	0.012 (4)	0.001 (3)
C12	0.055 (6)	0.026 (4)	0.031 (4)	0.004 (4)	0.014 (3)	0.003 (3)
C13	0.082 (7)	0.021 (4)	0.024 (4)	−0.003 (4)	0.007 (4)	−0.004 (3)
C14	0.075 (7)	0.030 (4)	0.024 (4)	−0.011 (4)	0.005 (4)	−0.004 (3)
C15	0.068 (7)	0.038 (5)	0.055 (5)	−0.003 (4)	0.007 (4)	0.000 (4)
C16	0.069 (7)	0.045 (5)	0.049 (5)	0.006 (4)	0.001 (4)	−0.009 (4)

### Geometric parameters (Å, °)

**Table:**

Br1—C1	1.927 (8)	C8—H8	0.9500
N1—C12	1.359 (11)	C9—C14	1.353 (11)
N1—C16	1.460 (10)	C9—C10	1.412 (10)
N1—C15	1.465 (11)	C10—C11	1.342 (11)
C1—C2	1.364 (10)	C10—H10	0.9500
C1—C6	1.391 (11)	C11—C12	1.443 (10)
C2—C3	1.408 (12)	C11—H11	0.9500
C2—H2	0.9500	C12—C13	1.408 (11)
C3—C4	1.392 (11)	C13—C14	1.371 (11)
C3—H3	0.9500	C13—H13	0.9500
C4—C5	1.393 (10)	C14—H14	0.9500
C4—C7	1.465 (11)	C15—H15A	0.9800
C5—C6	1.392 (11)	C15—H15B	0.9800
C5—H5	0.9500	C15—H15C	0.9800
C6—H6	0.9500	C16—H16A	0.9800
C7—C8	1.334 (12)	C16—H16B	0.9800
C7—H7	0.9500	C16—H16C	0.9800
C8—C9	1.472 (12)		
			
C12—N1—C16	120.5 (7)	C10—C9—C8	123.1 (7)
C12—N1—C15	123.1 (7)	C11—C10—C9	121.9 (8)
C16—N1—C15	115.9 (7)	C11—C10—H10	119.1
C2—C1—C6	124.3 (8)	C9—C10—H10	119.1
C2—C1—Br1	117.8 (6)	C10—C11—C12	122.7 (7)
C6—C1—Br1	117.9 (6)	C10—C11—H11	118.7
C1—C2—C3	117.4 (8)	C12—C11—H11	118.7
C1—C2—H2	121.3	N1—C12—C13	124.4 (7)
C3—C2—H2	121.3	N1—C12—C11	121.4 (7)
C4—C3—C2	121.9 (7)	C13—C12—C11	114.2 (8)
C4—C3—H3	119.0	C14—C13—C12	120.7 (7)
C2—C3—H3	119.0	C14—C13—H13	119.7
C3—C4—C5	116.8 (7)	C12—C13—H13	119.7
C3—C4—C7	120.7 (7)	C9—C14—C13	125.0 (7)
C5—C4—C7	122.4 (8)	C9—C14—H14	117.5
C6—C5—C4	123.8 (8)	C13—C14—H14	117.5
C6—C5—H5	118.1	N1—C15—H15A	109.5
C4—C5—H5	118.1	N1—C15—H15B	109.5
C1—C6—C5	115.7 (7)	H15A—C15—H15B	109.5
C1—C6—H6	122.2	N1—C15—H15C	109.5
C5—C6—H6	122.2	H15A—C15—H15C	109.5
C8—C7—C4	129.5 (8)	H15B—C15—H15C	109.5
C8—C7—H7	115.2	N1—C16—H16A	109.5
C4—C7—H7	115.2	N1—C16—H16B	109.5
C7—C8—C9	132.6 (8)	H16A—C16—H16B	109.5
C7—C8—H8	113.7	N1—C16—H16C	109.5
C9—C8—H8	113.7	H16A—C16—H16C	109.5
C14—C9—C10	115.4 (8)	H16B—C16—H16C	109.5
C14—C9—C8	121.3 (7)		
			
C6—C1—C2—C3	−2.3 (14)	C14—C9—C10—C11	4.5 (10)
Br1—C1—C2—C3	177.7 (6)	C8—C9—C10—C11	178.7 (7)
C1—C2—C3—C4	2.7 (13)	C9—C10—C11—C12	−1.8 (10)
C2—C3—C4—C5	−2.9 (12)	C16—N1—C12—C13	3.1 (11)
C2—C3—C4—C7	−178.7 (8)	C15—N1—C12—C13	173.9 (7)
C3—C4—C5—C6	2.8 (12)	C16—N1—C12—C11	−177.6 (7)
C7—C4—C5—C6	178.5 (8)	C15—N1—C12—C11	−6.8 (11)
C2—C1—C6—C5	2.1 (13)	C10—C11—C12—N1	179.5 (7)
Br1—C1—C6—C5	−177.8 (6)	C10—C11—C12—C13	−1.1 (10)
C4—C5—C6—C1	−2.4 (12)	N1—C12—C13—C14	−179.3 (7)
C3—C4—C7—C8	−142.8 (9)	C11—C12—C13—C14	1.3 (9)
C5—C4—C7—C8	41.7 (13)	C10—C9—C14—C13	−4.4 (10)
C4—C7—C8—C9	7.1 (15)	C8—C9—C14—C13	−178.8 (7)
C7—C8—C9—C14	−160.7 (9)	C12—C13—C14—C9	1.5 (11)
C7—C8—C9—C10	25.4 (12)		
